# Effects of single amino acid deficiency on mRNA translation are markedly different for methionine versus leucine

**DOI:** 10.1038/s41598-018-26254-2

**Published:** 2018-05-24

**Authors:** Kevin M. Mazor, Leiming Dong, Yuanhui Mao, Robert V. Swanda, Shu-Bing Qian, Martha H. Stipanuk

**Affiliations:** Division of Nutritional Sciences, Cornell University, Ithaca, NY 14853 USA

## Abstract

Although amino acids are known regulators of translation, the unique contributions of specific amino acids are not well understood. We compared effects of culturing HEK293T cells in medium lacking either leucine, methionine, histidine, or arginine on eIF2 and 4EBP1 phosphorylation and measures of mRNA translation. Methionine starvation caused the most drastic decrease in translation as assessed by polysome formation, ribosome profiling, and a measure of protein synthesis (puromycin-labeled polypeptides) but had no significant effect on eIF2 phosphorylation, 4EBP1 hyperphosphorylation or 4EBP1 binding to eIF4E. Leucine starvation suppressed polysome formation and was the only tested condition that caused a significant decrease in 4EBP1 phosphorylation or increase in 4EBP1 binding to eIF4E, but effects of leucine starvation were not replicated by overexpressing nonphosphorylatable 4EBP1. This suggests the binding of 4EBP1 to eIF4E may not by itself explain the suppression of mRNA translation under conditions of leucine starvation. Ribosome profiling suggested that leucine deprivation may primarily inhibit ribosome loading, whereas methionine deprivation may primarily impair start site recognition. These data underscore our lack of a full understanding of how mRNA translation is regulated and point to a unique regulatory role of methionine status on translation initiation that is not dependent upon eIF2 phosphorylation.

## Introduction

The effects of amino acid availability on the regulation of protein synthesis are believed to be mediated predominantly through the mechanistic target of rapamycin complex 1 (mTORC1) pathway, which appears to be especially sensitive to leucine availability^1,2^, and through regulation of the phosphorylation status of the alpha subunit of eukaryotic initiation factor 2 (eIF2)^3^.

A major downstream target of mTORC1 signaling is the eukaryotic initiation factor 4E binding proteins (4EBP1/2). Most research has been conducted on 4EBP1, but 4EBP2 appears to function similarly. 4EBP competes with eIF4G for binding to eIF4E^4–7^. When eIF4E is bound to 4EBP, eIF4E recognizes the 5′ cap but cannot recruit eIF4G^4–7^. Effects of mTORC1 signaling on translation initiation are usually attributed to its phosphorylation of 4EBP^4,8–12^. Under nutrient-sufficient conditions mTORC1 phosphorylates 4EBP at several sites, which inhibits 4EBP binding to eIF4E and promotes cap-dependent translation. Conversely, mTORC1 is inhibited under conditions such as low insulin or low amino acids, and 4EBP becomes hypophosphorylated and bound to eIF4E, thus inhibiting translation^4,8–12^. Leucine is particularly effective in activating mTORC1^2,13,14^, and both Sestrin2^1^ and leucyl-tRNA synthetase^15^ have been identified as cytosolic leucine sensors that promote mTORC1 localization to the lysosomal membrane and activation of mTORC1 kinase activity when they are in their leucine-bound forms.

Translation initiation is also regulated in response to amino acids by phosphorylation of the alpha subunit of eIF2. When an essential amino acid is limiting, its tRNA(s) cannot be fully aminoacylated and the concentration(s) of the non-aminoacylated (“uncharged”) tRNA(s) increase(s)^16^. Uncharged tRNAs bind to the histidyl-tRNA synthetase−related regulatory domain of GCN2 (i.e., eIF2α kinase 4), causing its activation^17–21^. Active GCN2 then phosphorylates the alpha subunit of eIF2^22–24^. eIF2 functions in its GTP-loaded form to form the 43S preinitiation complex (PIC) with initiator methionine tRNA (Met-tRNA_i_^Met^), the 40S ribosomal subunit, eIF3, eIF1 and eIF1A. The engagement of the PIC with a start codon results in hydrolysis of eIF2-GTP to eIF2-GDP and the release of eIF2-GDP. When eIF2 is phosphorylated, it inhibits the guanine nucleotide exchange factor eIF2B and blocks the recycling of eIF2-GDP to eIF2-GTP. With less eIF2-GTP, there is less formation of active 43S PIC, and mRNA translation initiation is suppressed^25,26^.

Despite a number of studies looking at the effects of starvation of total amino acids, few studies have compared the effects of deficiencies of different single essential amino acids on protein synthesis. In rats and mice, single essential amino acid deprivation can affect hepatic polysome profiles^27–30^, rates of hepatic protein synthesis^30,31^, and the phosphorylation status of 4EBP1^27,30–32^ and eIF2^29–31,33–36^. Studies in hepatocytes or HeLa cells demonstrated effects of short-term starvation of single amino acids on protein synthesis, polysome formation, and PIC formation^37,38^. Current evidence suggests that the relative roles of the GCN2 and mTORC1-mediated responses to amino acid starvation may differ depending on the specific amino acid(s) that is (are) deficient and the particular tissue^31,39–41^. Mice treated with asparaginase to lower asparagine and glutamine concentrations had reduced rates of protein synthesis in liver and spleen, which was accompanied by an increase in eIF2 phosphorylation in both tissues but by a decrease in 4EBP1 hyperphosphorylation only in liver^41^. Studies in bovine mammary tissue slices and MAC-T cells demonstrated a significant effect of leucine but not of other essential amino acids on eIF2 phosphorylation and mTORC1 kinase actions^42^. In a study in isolated rat hepatocytes, deprivation of methionine had a greater inhibitory effect on protein synthesis than did deprivation of other essential amino acids^37^. In this paper, we further explore effects of single essential amino acid deprivation on 4EBP1 and eIF2 phosphorylation status and protein synthesis.

## Results

To evaluate the effects of single amino acid deprivation on cellular 4EBP1 and eIF2 phosphorylation and protein synthesis in HEK293T cells, we chose a time point of 12 h after the switch from complete to amino acid-deficient medium. This time period allowed both sufficient depletion of the intracellular essential amino acid pool of the limiting amino acid to impact mRNA translation as well as time for cells to recover from the integrated stress response that is often observed in response to a change in culture medium.

### Leucine and methionine deprivation strongly inhibit translation initiation and polysome formation

**Figure 1:**
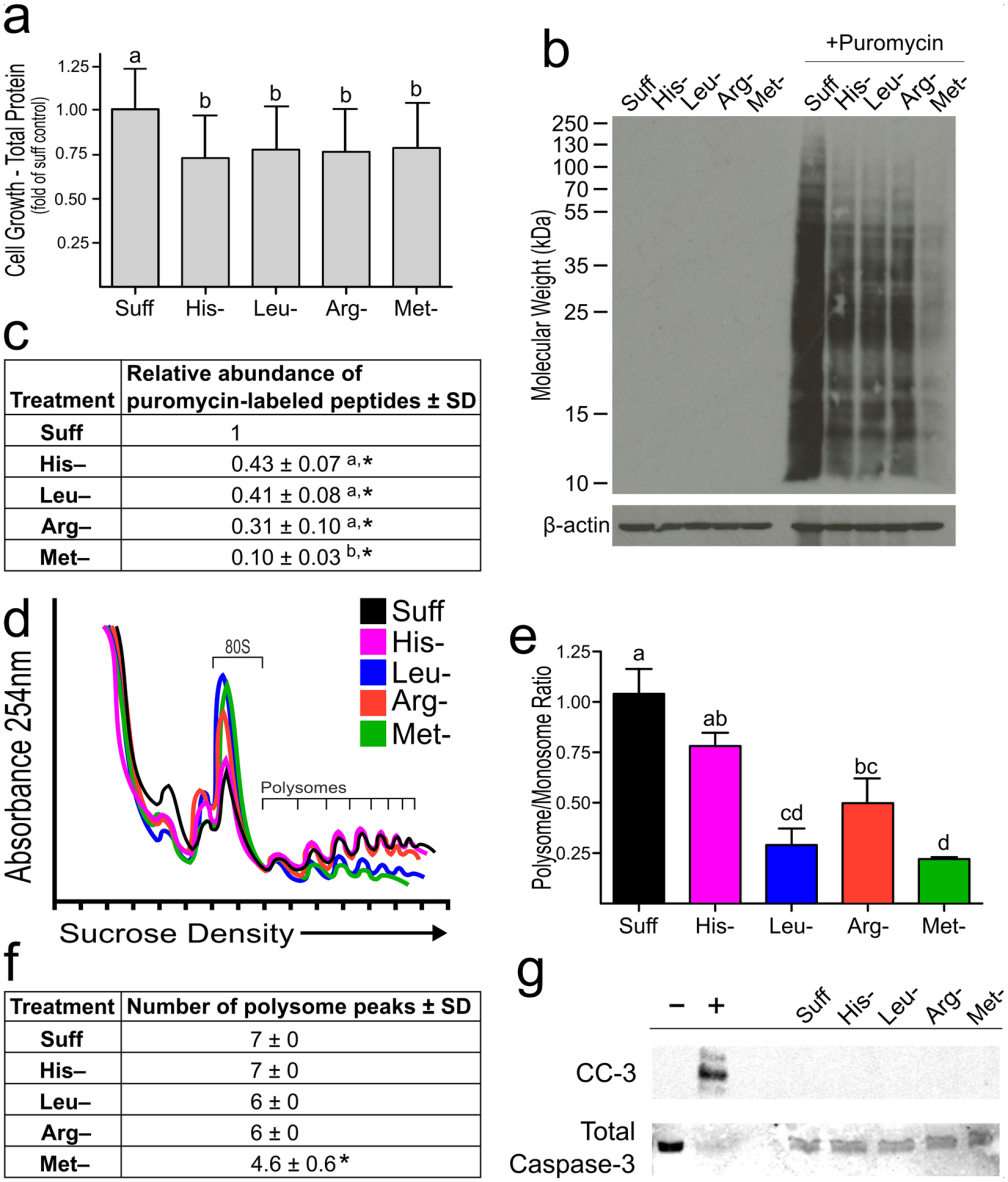
Changes in the growth, peptide translation and polysome profile of HEK293T cells in response to deficiency of a single essential amino acid. Cells were grown in complete medium (Suff) or in medium deficient in histidine (His‒), arginine (Arg‒), leucine (Leu‒) or methionine (Met‒) for 12 h. Values are means ± SD for 3 separate experiments. Results in (**a** and **c**‒**g**) are from one set of experiments, and those in b are from a separate set of experiments. Bars labeled with different letters are significantly different by least squares analysis with Tukey’s post hoc test at p ≤ 0.05. Values labeled with an asterisk (*) had a 95% confidence interval that did not include 1.0, making values significantly different from the Suff control at p ≤ 0.05; when relative values for amino acid-deficient conditions were analyzed, the replicate number had no significant effect in the overall model. (**a**) Final protein content of cells grown in amino acid sufficient or deficient medium for 12 h, expressed as a fraction of the final protein content of cells grown in sufficient medium. (**b**) Representative western blot showing rate of mRNA translation as assessed by puromycin labeling of newly synthesized proteins. (**c**) Relative densities of puromycin-labeled peptides. The density for the Suff condition was set at 1.0 for each blot (i.e., each separate experiment). (**d**) Image of overlaid polysome profiles from a representative experiment, illustrating differences in polysome profiles. (**e**) Ratios of polysome area to monosome area of polysome profiles. (**f**) Number of peaks in the polysome fraction of the polysome profiles. (**g**) Cleaved caspase 3 assay on 293 T cells. A representative western blot for the cleaved caspase 3 assay; the negative (−) and positive (+) control extracts are Jurkat cells treated without (−) or with (+) cytochrome c.

Deprivation of arginine, histidine, leucine or methionine each resulted in an ~25% decrease in cell growth/proliferation as measured by total protein content of the cultured cells compared to cells grown in sufficient medium. The extent of reduction in total protein was similar for cells starved for any one of the four tested amino acids (Fig. 1a).

Somewhat surprisingly, the rate of mRNA translation as assessed by puromycin labeling of newly synthesized proteins showed a much more adverse effect of methionine deficiency than of histidine, leucine or arginine deficiency (Fig. 1b,c). Puromycin is a Tyr-tRNA mimetic that terminates translation by ribosome-catalyzed covalent incorporation into the nascent polypeptide chain^43,44^. Puromycin labeling was reduced by about 60% for cells deprived of histidine, leucine or arginine but by 90% for cells deprived of methionine. Because the puromycin labeling experiment captured mRNA translation at only the 12-h time point whereas the total protein content reflects growth over the entire 12-h period, the apparent more severe effect of amino acid starvation on protein synthesis than on cell growth could reflect a progressively more severe effect of amino acid starvation, especially methionine starvation, on protein synthesis with increased culture time. Alternatively, the more modest effects on cell growth than on protein synthesis could be explained by a compensatory suppression of protein degradation.

Starvation of each of the four tested amino acids had a different effect on the polysome profile (Fig. 1d). Histidine deprivation did little to change the polysome profile. Arginine deprivation resulted in an increase in the monosome peak and a slight decrease in the polysome fractions. Leucine deprivation resulted in an increase of the monosome peak and an even larger reduction in the polysome fractions compared to histidine or arginine deprivation. Methionine deprivation resulted in an increase in the monosome peak and the most extensive decrease in polysome fractions. The shift in polysome profiles was quantified as the ratio of the area of the polysome peaks (P) to the area of the monosome peak (M). As shown in Fig. 1e, histidine starvation had no effect on the P/M ratio. Arginine significantly reduced the P/M ratio compared to that for cells grown in sufficient medium or for cells deprived of histidine. Leucine and methionine deprivation had the most drastic effects on the P/M ratio, with the P/M ratios being significantly lower than those of cells grown in histidine- or arginine-deficient medium or in sufficient medium. The observed changes in the P/M ratios were mimicked by the number of peaks observed in the polysome fractions. Methionine deprivation resulted in a decrease in polysome peak number from 7 under sufficient conditions to 4.7 ± 0.6 (SD), whereas leucine and arginine deprivation only decreased polysome peak number from 7 to 6 and histidine had no effect (Fig. 1f).

Thus, although deprivation of any one of the four amino acids tested had a similar inhibitory effect on cell growth/proliferation over the 12-h period of culture, deprivation of the different amino acids had varying effects on measures of mRNA translation at the 12-h time point. In particular, the effectiveness of methionine deprivation in suppressing polysome formation and protein synthesis was striking with methionine deficiency reducing both the number of polysomes and the synthesis of puromycin-labeled proteins more markedly than a deficiency of histidine, leucine or arginine. To determine if methionine deprivation might be sufficiently detrimental to the cell to induce apoptosis, we analyzed cells for the presence of cleaved caspase 3. However, there was no increase in cleaved caspase 3 as a result of a deprivation of any amino acid tested (Fig. 1g).

### Leucine but not arginine, histidine or methionine deprivation results in an increase in 4EBP1‒eIF4E association

**Figure 2:**
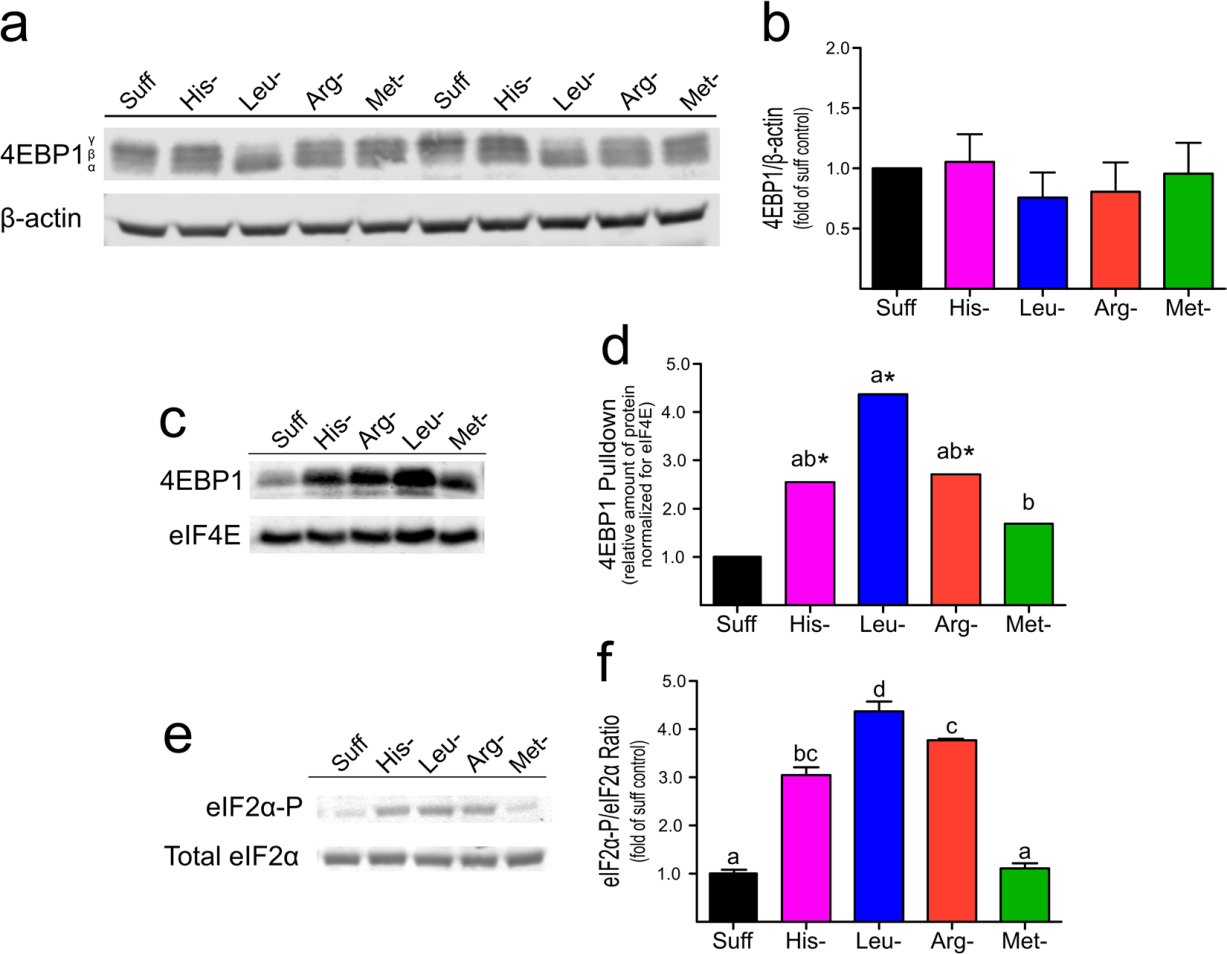
Differences in 4EBP1 and eIF2 phosphorylation in HEK293T cells in response to deficiency of a single essential amino acid. Cells were grown in complete medium (Suff) or in medium deficient in histidine (His–), arginine (Arg–), leucine (Leu–) or methionine (Met–) for 12 h. Values are means ± SD for 3 separate experiments. All results (**a**‒**f**) are from the same set of experiments. Bars labeled with different letters are significantly different by least squares analysis with Tukey’s post hoc test at p ≤ 0.05. Values labeled with an asterisk (*) had a 95% confidence interval that did not include 1.0, making values significantly different from the Suff control at p ≤ 0.05; when relative values for amino acid-deficient conditions were analyzed. (**a**) A representative western blot of 4EBP1 in the lysis solution used for the eIF4E pulldown assays. (**b**) Relative abundance of total 4EBP1 expressed as fold the value for cells cultured in complete medium in the same replicate experiment after normalization for abundance of β-actin. (**c**) A representative western blot for the amount of 4EBP1 pulled down with eIF4E. (**d**) Amount of 4EBP1 associated with eIF4E expressed as fold the mean value for cells cultured in complete medium after normalization to eIF4E. (**e**) A representative western blot for phosphorylated and total eIF2α. (**f**) Ratio of phosphorylated eIF2α to total eIF2α expressed as fold the mean value for cells cultured in complete medium.

Because 4EBP1 phosphorylation status is thought to regulate the rate of cap-dependent mRNA translation in response to mTORC1 signaling, the effect of single amino acid deprivation on 4EBP1 phosphorylation status was determined (Fig. 2a). The relative abundance of total 4EBP1 was not significantly different for cells cultured in amino acid-deficient medium than in cells cultured in complete medium (Fig. 2b), but the hyperphosphorylation of 4EBP1 was markedly lower in cells cultured in leucine-deficient medium than in cells cultured in control or histidine-, arginine- or methionine-deficient medium (Fig. 2a), suggesting that mTORC1-mediated phosphorylation of 4EBP1 is more sensitive to leucine than the other tested amino acids.

To determine if the changes in 4EBP1 phosphorylation state corresponded to changes in 4EBP1 binding to cap-associated eIF4E, eIF4E was pulled down using a cap analog, and the relative amount of 4EBP1 bound to eIF4E was determined (Fig. 2c). Compared to cells cultured in sufficient medium, the amount of 4EBP1 pulled down with eIF4E (normalized for the amount of eIF4E) was significantly greater (P ≤ 0.05) for cells cultured in histidine-, leucine- or arginine-deficient medium but not for those cultured in methionine-deficient medium (Fig. 2d). The observation that the lowest apparent increase in 4EBP1 binding occurred in methionine-deficient cells whereas the most robust increase occurred in leucine-deficient cells is consistent with the greater effect of leucine deficiency on the degree of 4EBP1 phosphorylation. Therefore, although methionine deprivation was more effective than leucine deprivation at suppressing polysome formation and mRNA translation, methionine deprivation was much less effective than leucine deprivation at promoting 4EBP1 dephosphorylation and 4EBP1 binding to eIF4E.

Because amino acid deprivation can inhibit mRNA translation independently of mTORC1 through the phosphorylation of eIF2, we measured the extent of eIF2 phosphorylation under the different culture conditions. As can be seen in Fig. 2e,f, deprivation of leucine, histidine or arginine resulted in significant increases in eIF2 phosphorylation whereas deprivation of methionine had no effect on eIF2 phosphorylation compared to cells grown in sufficient medium. Thus, eIF2 phosphorylation was not associated with the suppressed protein synthesis observed in response to methionine deprivation, similar to the lack of change in 4EBP1 phosphorylation status and association with eIF4E in methionine-deprived cells.

### Expression of a nonphosphorylatable 4EBP1 mutant does not inhibit translation initiation and polysome formation

**Figure 3:**
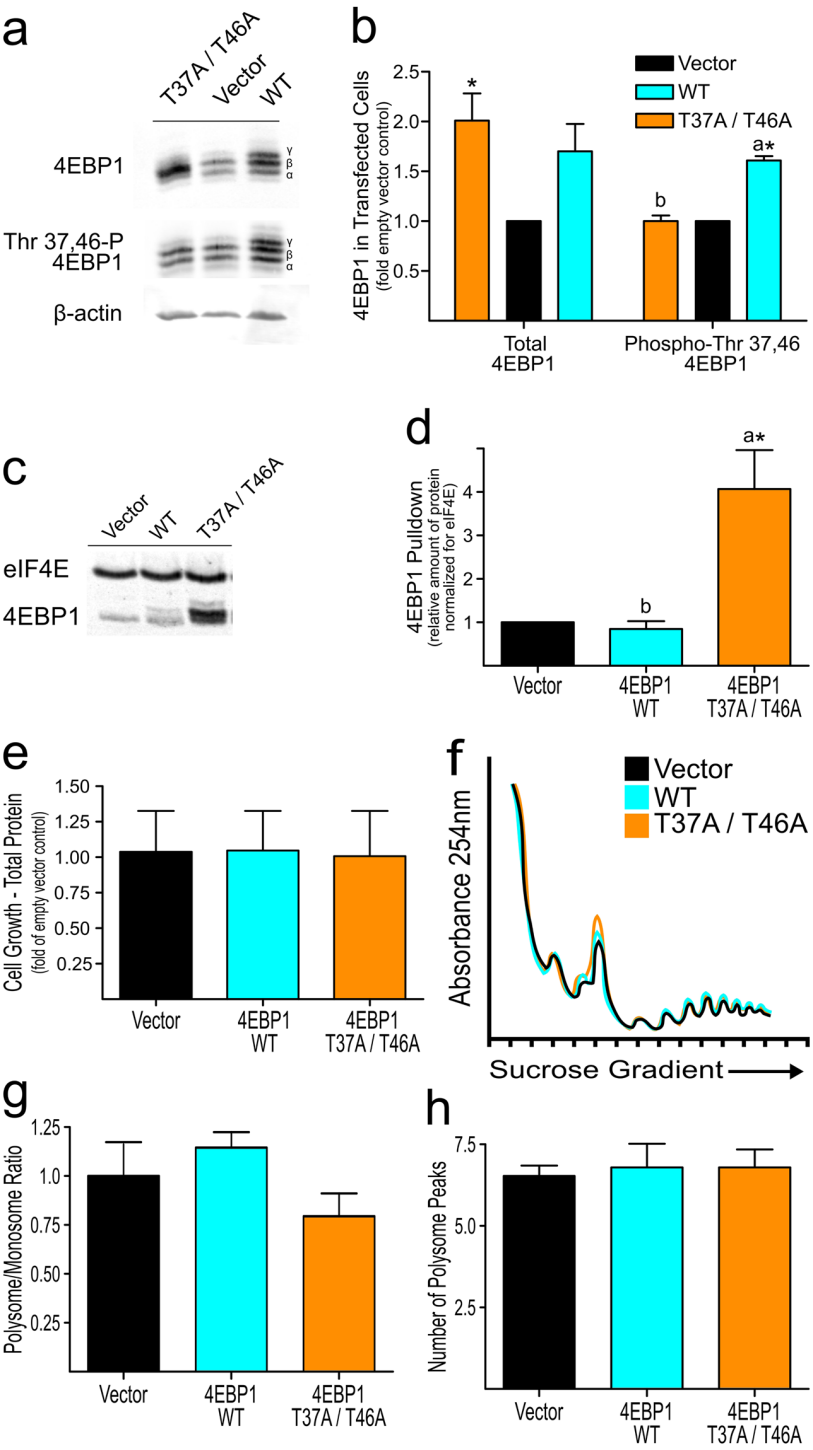
Effect of transfection of HEK293T cells with wild-type versus constitutively active 4EBP1. Cells were transfected with empty vector, wildtype 4EBP1, or constitutively active mutant 4EBP1(T37A/T46A) and grown in sufficient control medium for 24 h. Values are means ± SD for 3 separate experiments. All results (**a**‒**h**) are from the same set of experiments. Values for separate experiments were normalized by setting the vector control value at 1.0. Bars labeled with different letters are significantly different by least squares analysis with Student’s post hoc test at p ≤ 0.05. Values labeled with an asterisk (*) had a 95% confidence interval that did not include 1.0, making values significantly different from the vector control at p ≤ 0.05. (**a**) Representative western blot for abundance of total and Thr 37,46-phosphorylated 4EBP1 in transfected cells. (**b**) Abundance of total and Thr 37,46-phosphorylated 4EBP1, normalized for actin and expressed as fold the mean values for cells transfected with empty vector. (**c**) Representative western blot for m7 cap-analog pulldown of eIF4E and associated 4EBP1 from transfected cells. (**d**) Relative amount of 4EBP1 associated with eIF4E, expressed as fold the mean value for cells transfected with empty vector after normalization to eIF4E. (**e**) Cell growth assessed by total protein content at 24 h post-transfection, expressed as a percentage of total protein in cells transfected with the empty vector. (**f**) Representative image of polysome profiles for transfected cells. (**g**) Ratios of polysome to monosome areas of polysome profiles. (**h**) Number of peaks in the polysome fraction of the polysome profiles.

To further elucidate the effect of 4EBP1 binding to cap-associated eIF4E on the polysome profile, we expressed constitutively active 4EBP1 with Ala residues replacing the Thr37 and Thr46 residues. Transfection of HEK293T cells with wild-type 4EBP1 or mutant constitutively active 4EBP1(T37A/T46A) plasmids tended to result in higher cellular levels of total 4EBP1 protein at 24 h post-transfection compared to control cells transfected with vector alone, but only that for cells transfected with the T37A/T46A mutant plasmid was significantly greater than the vector control (Fig. 3a,b). However, the amount of phosphorylated 4EBP1 detected by an antibody specific for the phosphorylated Thr37 and Thr46 sites of 4EBP1 indicated an increase in phosphorylated 4EBP1 only in cells transfected with the wild-type 4EBP1. The increase in the hyperphosphorylated (gamma) band for 4EBP1 in cells transfected with wild-type but not in those transfected with 4EBP1(T37A/T46A) is consistent with the phosphorylation of 4EBP1 by mTORC1 (on Thr70 and Ser65) being dependent upon the prior phosphorylation of Thr37 and Thr46. Phosphorylation of all four residues is required to inactivate 4EBP1′s ability to bind eIF4E^11^.

Because hyperphosphorylation of 4EBP1 by mTORC1 blocks its association with eIF4E, the greater amount of nonphosphorylatable 4EBP1 in cells transfected with 4EBP1(T37A/T46A) would be expected to result in more association of 4EBP1 with eIF4E. Assuming that overexpression of 4EBP1 had no dramatic effect on the abundance of other eIF4E binding proteins (i.e., 4EBP2, 4EBP3, eIF4G1 or eIF4G2), this was indeed the case, as shown in Fig. 3c,d. Transfection of cells with wild-type 4EBP1 had no effect on the amount of eIF4E-associated 4EBP1 compared to empty vector-transfected control cells, but the eIF4E pulled down from cells transfected with mutant 4EBP1(T37A/T46A) had 4-times as much bound 4EBP1. This confirms that the mutant constitutively active 4EBP1(T37A/T46A) bound eIF4E efficiently even when cells were grown in complete medium. These results are consistent with the increased binding of 4EBP1 to eIF4E that accompanied the decreased hyperphosphorylation of 4EBP1 observed in cells cultured in leucine-deficient medium (Fig. 2a,c,d).

At 24 h after transfection, there was no significant difference in the total amount of protein in the various cultures, suggesting little effect of increased 4EBP1 binding to eIF4E on the rate of cell growth/proliferation, at least in cells cultured in complete medium (Fig. 3e). Overlay of polysome profiles for cells transfected with empty vector, wild-type 4EBP1, or 4EBP1(T37A/T46A) demonstrated a slight increase in the monosome peak in cells transfected with the mutant nonphosphorylatable 4EBP1 (Fig. 3f). Calculation of P/M ratios and the number of polysome peaks, however, demonstrated no significant reduction for cells transfected with 4EBP1(T37A/T46A) compared to values for cells transfected with the empty vector (Fig. 3g,h). As in the experiments shown in Fig. 1, these studies with 4EBP1(T37A/T46A) suggest that an increase in 4EBP1 association with eIF4E by itself does not inhibit cell growth and has relatively little effect on the formation of polysomes. This suggests that a change in 4EBP1 phosphorylation/eIF4E binding status that is unaccompanied by other changes may have little effect on overall mRNA translation.

### Leucine and methionine inhibit translation through different mechanisms

**Figure 4:**
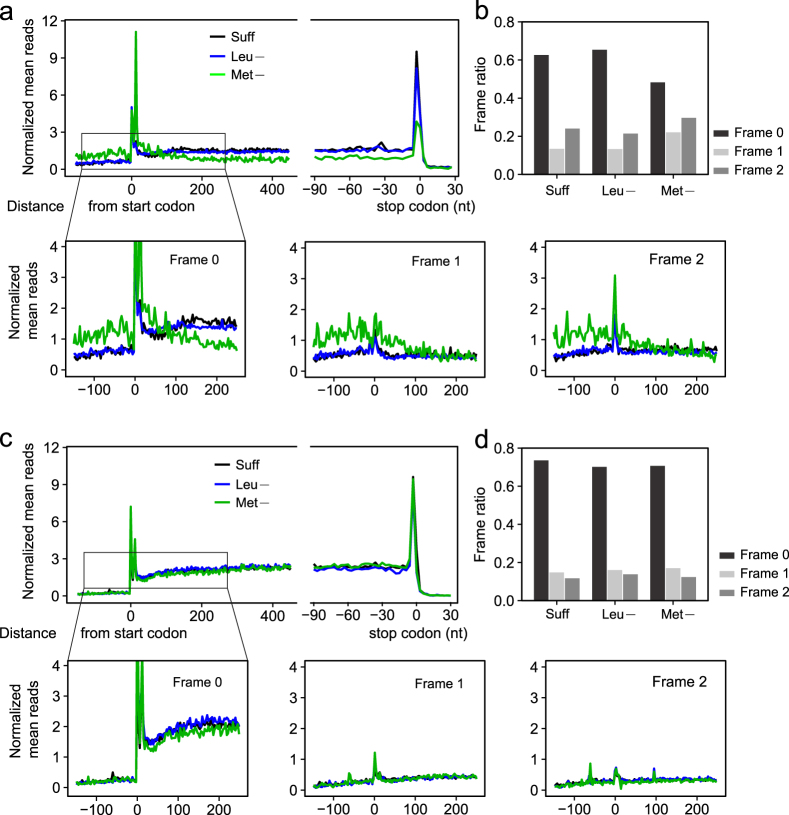
Ribosome profiling in response to deficiency of single essential amino acids. (**a**) Metagene analysis of ribosome density in HEK293T cells grown in complete medium or in medium deficient in leucine or methionine for 12 h. The density of ribosome footprints mapped to the human transcriptome are determined at single nucleotide positions and averaged across transcripts aligned at start and stop codons. The zoomed in region shows the read distribution by reading frame. (**b**) Fractional distribution of ribosome footprints mapped to the coding regions by reading frame using Ribo-seq data obtained in the presence of cycloheximide. (**c**) Metagene analysis of ribosome density in HEK293T cells prepared in the absence of cycloheximide. (**d**) Fractional distribution of ribosome footprints mapped to the coding regions by reading frame, using Ribo-seq data obtained in the absence of cycloheximide.

To further elucidate the differences observed in polysome profiles due to deprivation of different single essential amino acids, we performed genome-wide ribosome profiling on HEK293T cells grown in sufficient, leucine-deficient or methionine-deficient medium. The ribosome density at each position was first normalized by the length of each mRNA, and then the normalized reads were aggregated by aligning all mRNAs by their distance from the start codon using both in-frame (frame 0) and out-of-frame [frame 1 (+1) and frame 2 (+2)] codon positioning. Aggregated results were divided by the number of genes (mRNAs) that were identified in the global ribosome profiling and thus are reported in Fig. 4 as the mean ribosome density at each codon position.

Looking at the frame 0 results for polysomes collected with cycloheximide pretreatment (Fig. 4a), leucine deficiency did not reduce mean ribosome densities at positions downstream of the start codon, whereas methionine deprivation markedly decreased mean ribosome densities downstream of the start codon except for an increased density at a point 12 nt downstream of the start codon. Methionine deprivation also resulted in an increase in ribosome densities at positions upstream of the start codon, which contrasted with a low abundance of upstream ribosomes in cells cultured in sufficient or leucine-deficient medium. The peak at 12 nt downstream from the start codon, which was observed under all conditions, has previously been characterized^44^ and has been attributed to post-initiation pausing of ribosomes in what is hypothesized to be an obligatory quality control step between initiation and elongation that determines whether translation proceeds or is abandoned. The lack of ribosome densities downstream of mRNA start codons in methionine-deficient cells is consistent with the drastic suppression of polysome formation observed in methionine-deficient cells (Fig. 1b,c,d).

To determine if methionine- or leucine-deprivation results in errors in start site recognition, the ribosome densities at out-of-frame codons were also determined. As can be seen in Fig. 4a, methionine-, but not leucine-deprivation, resulted in an increase in mean ribosome densities both upstream and downstream of the start codon in frame 1 and frame 2 when compared to cells grown in sufficient medium. This was further analyzed by determining the percentage of active ribosomes in each frame (Fig. 4b). Leucine deprivation did not increase the percentage of ribosomes in frames 1 and 2 compared to cells grown in sufficient medium (35% vs 38%). In contrast, methionine deprivation substantially increased the percentage of ribosomes in frames 1 and 2 (52% vs 38%, chi-squared test, *P* < 2.2 × 10^−16^). The increased out-of-frame translation under methionine starvation is likely due to the shortage of initiator tRNA at the annotated start codons, which results in the scanning ribosomes selecting non-canonical start codons for initiation. Notably, conducting ribosome profiling in the presence of cycloheximide helps capture early translation events.

To demonstrate that the increased ribosome densities in the 5′UTR represent true translation events, we repeated the ribosome profiling experiments without cycloheximide (Fig. 4c,d). The subsequent ribosome runoff is expected to deplete 5′UTR translation, especially the ultra-short open reading frames. This was indeed the case. The increased 5′UTR translation after methionine deprivation was no longer evident (Fig. 4c). As expected, more ribosomes accumulated toward the end of coding regions, including at the stop codon. Ribosome densities downstream of the start codon were still lower under no cycloheximide conditions in methionine starved cells than in cells cultured in sufficient or leucine deficient medium (Fig. 4c). The lowered in-frame rate (or frame 0 ratio) after methionine starvation is still discernible in the absence of cycloheximide (Fig. 4c). Collectively, these results suggest that methionine-deficiency likely interferes with the ability of ribosomes to recognize the correct start site.

## Discussion

Cells require an exogenous source of the amino acids they cannot synthesize. For mammalian cells, this includes those that cannot be synthesized by the body (e.g., histidine, methionine and leucine) as well as amino acids that cannot be synthesized by the particular cell (e.g., arginine). Although arginine can be synthesized in the body via sequential contributions of the intestine and kidney, the need for arginine cannot be met by endogenous synthesis in most cell types. Starvation of cells for any amino acid they cannot synthesize results in a decrease in cell growth and proliferation. The roles of amino acids as substrates and regulators of protein synthesis are well-established. Nevertheless, our comparison of the effects of different amino acids on various measures of mRNA translation and various mechanisms of regulation of mRNA translation yielded several unanticipated findings.

Although the lack of any one of the four amino acids we tested resulted in a similar overall restriction of cell growth, effects on polysome formation ranged from negligible in the case of histidine deficiency to moderate in the case of arginine deficiency to severe in the case of leucine or methionine deficiency. Puromycin labeling showed diminished protein synthesis in cells cultured in medium deficient in any one of the four amino acids tested, but protein synthesis was significantly more diminished in cells cultured in methionine-deficient medium than in cells cultured in medium deficient in leucine, arginine or histidine. Of the conditions tested, only leucine deprivation resulted in 4EBP1 dephosphorylation and enhanced 4EBP1 binding to eIF4E. In contrast, phosphorylation of eIF2 was elevated in cells cultured in histidine-, leucine- or arginine-deficient medium but not in cells cultured in methionine-deficient medium. The phosphorylation state of 4EBP1 or eIF2 did not closely correlate with puromycin labeling or polysome profiles across the different conditions, and the effects of methionine starvation did not appear to be explained by either 4EBP1 dephosphorylation or eIF2 phosphorylation. Collectively, these observations underscore our incomplete understanding of translation regulation by amino acids and suggest that the presence or absence of particular amino acids may exert effects via different mechanisms.

Contrary to prevailing views that 4EBP1 is a strong regulator of mRNA translation^10,45–47^, our results do not support an overarching role of 4EBP1 binding to eIF4E in suppressing global mRNA translation in amino acid deficiency. Although leucine deprivation strongly inactivated mTORC1’s kinase activity as judged by 4EBP1 dephosphorylation, the resulting increase in 4EBP1’s binding affinity for eIF4E may not explain the effect of leucine deprivation on polysome formation because the effect could not be reproduced by expression and binding of mutant 4EBP1(T37A/T46A) to eIF4E. Furthermore, the observation that methionine, histidine or arginine deprivation had no significant effect on the binding of 4EBP1 to eIF4E, in contrast to the marked effect of leucine deficiency, challenges the notion that 4EBP1 binding to eIF4E is a major suppressor of mRNA translation in response to single amino acid deprivation. The observation that leucine deprivation had a much larger effect on 4EBP1‒eIF4E association than did methionine, histidine or arginine deprivation, however, is consistent with leucine exerting somewhat unique effects through regulation of mTORC1 kinase activity. It is possible that leucine’s effects on mRNA translation/polysome formation is due to the action of some other protein that also is regulated by mTORC1 activity, and this could include proteins that act to regulate either initiation or elongation. A different target of mTORC1, such as S6K1 or its downstream targets eIF3 and eIF4B, theoretically could have a global effect on mRNA translation^48–50^. A global effect of these mTORC1 targets on mRNA translation has not been shown, however. Another possibility is that leucine and its effects on mTORC1/4EBP1 could selectively promote translation of a subset of mRNAs (e.g TOP-mRNAs), with leucine deficiency resulting in reduced translation of one or more members of this subset to effect changes in gene expression that indirectly mediate a more global suppression of mRNA translation^51–53^. Regardless of these possibilities, our data strongly indicate that an increase in hypophosphorylated 4EBP1 capable of binding to eIF4E is not sufficient to suppress global mRNA translation and also is unlikely to be the major mechanism by which most amino acids regulate translation. Similar to our findings, a study in isolated rat cardiomyocytes demonstrated a lack of effect of 4EBP1 binding to eIF4E on overall protein synthesis^40^.

Our observation is at odds with past studies in which transfection of cells with a constitutively active 4EBP1 mutant inhibited the translation of cap-dependent genes. These studies, however, measured the reduction of translation through reporter gene constructs containing 5ʹUTRs from known cap-dependent genes^4,54,55^. Our observations are consistent with several reports of a lack of association of 4EBP1 phosphorylation and total protein synthesis. Expression of a nonphosphorylatable 4EBP1 in rat Rat1a and TGR cells resulted in no change in total protein synthesis in either cell type, and a change in the polysome profile was observed only in the TGR cells^56^. In insulin deprived-L6-myoblasts, leucine deprivation but not histidine deprivation reduced 4EBP1 hyperphosphorylation and increased 4EBP1 association with eIF4E, whereas both leucine deprivation and histidine deprivation decreased protein synthesis and decreased eIF2B activity^39^. In addition, when insulin was added back protein synthesis and eIF2B activity were still inhibited under amino acid deprivation but leucine deprivation no longer affected 4EBP1phosphorylation and association with eIF4E. These findings resulted in the conclusion that eIF2 phosphorylation/eIF2B exchange activity but not 4EBP1 hypophosphorylation/4EBP1 binding to eIF4E was the primary mediator of a decrease in protein synthesis in response to leucine- or histidine-deprivation^39^. Our observation that eIF2 phosphorylation was not significant in cells cultured in methionine-deficient medium, however, suggests that the eIF2 guanine nucleotide exchange mechanism may not explain responses to deficiencies of all amino acids, at least not to a deficiency of methionine. Thus, the overall contributions of 4EBP1and eIF2 phosphorylation status on the control of mRNA translation remain uncertain, but the different responses observed to deficiencies of individual amino acids in this study suggest that different amino acids, at least leucine and methionine, act via different mechanisms. The study of cells exposed to deficiencies of different individual amino acids may be a fruitful approach to clarifying the contributions of different mechanisms.

Comparison of the effects of leucine deprivation and methionine deprivation on ribosome profiles provides further insights into potential mechanisms involved in the suppression of mRNA translation by leucine versus methionine deficiency. Leucine deprivation did not change the overall shape of the global ribosome density profile compared to that for cells cultured in sufficient medium, but it did result in slightly lower densities of ribosomes in the aggregated open reading frames. This suggests that leucine deprivation may inhibit the loading of ribosome onto mRNA, leading to an overall decrease in the number of ribosomes on mRNA, but likely has little effect on the movement of ribosomes along the mRNA during the translation process, resulting in a comparable distribution of ribosomes along the mRNA in leucine-deficient and sufficient conditions. This conclusion is consistent with the appearance of the polysome profiles for cells deprived of leucine. Leucine-deficient cells exhibited a lower P/M ratio compared to observations for cells grown in sufficient medium, but the number of peaks was only minimally affected. These observations indicate that many mRNAs are still readily translated under leucine-deficient conditions.

In contrast to the ribosome profiling results for leucine-deficient cells, the global distribution of ribosomes on mRNA under conditions of methionine deficiency appeared to be random, with many ribosomes associated with regions upstream of the start codons and with many ribosomes appearing to be out-of-frame with the coding sequence. This suggests that under methionine deprivation the correct start site cannot be recognized, leading to a high number of ribosomes initiating both in the 5′UTR and out of frame. The fact that the heavier polysome fractions were absent in polysome profiles of cells cultured in methionine-deficient medium suggests that many ribosomes may fall off the mRNA shortly after they incorrectly initiate. This would be consistent with the ‘ambush hypothesis^57–59^, which claims that mRNAs contain a large number of out-of-frame stop codons to minimize out of-frame translation as a result of frameshift mutations. This hypothesis is supported by the dramatic decrease in puromycin-labeled peptides observed in methionine-deficient cells.

Ribosome profiles generally look different when done with or without cycloheximide. As is typically observed for a comparison of profiles done with/without cycloheximide, our repeat of the experiment without cycloheximide resulted in a shift of the ribosomes toward the stop codon, indicating that the ribosomes continue to move when not fixed in place by cycloheximide treatment. The lack of ribosomes associated with the 5′UTR in methionine-deficient cells when cycloheximide was not present, in contrast to the case when cycloheximide was present, supports the notion that the increased ribosome densities in the 5′UTR in the presence of cycloheximide represent true translation events. Methionine-deficient cells exhibited lower frame 0 ribosome binding in both the absence and presence of cycloheximide, consistent with the hypothesis that methionine starvation interferes with correct start site recognition.

The apparent ability of methionine deficiency to interfere with start site recognition suggests that methionine may play a unique role in regulating translation initiation. Methionine-specific effects could be mediated by a lack of methionine-charged initiator tRNA or by a lack of metabolites of methionine, such as *S*-adenosylmethionine (SAM) or cysteine. SAM is the donor of methyl groups to numerous biological reactions^60^. Cysteine is substrate for the synthesis of glutathione, which plays a critical role in maintaining redox balance and preventing oxidative stress. Our methionine-deficient medium contained cysteine so a lack of cysteine/glutathione is not a likely explanation of our findings, but either a lack of methionine-charged tRNA_i_^Met^ or a lack of SAM for methylation reactions is a possible explanation.

It is highly probable that after 12 h of exposure of cells to methionine-deficient medium, both methionine initiator- and elongator-tRNA aminoacylation levels would have decreased, and the decrease in Met-tRNA_i_^Met^ levels due to methionine deprivation could inhibit formation of the 43S PIC^23^. Met-tRNA_i_^Met^ binding to eIF2-GTP is facilitated by the presence of methionine on the tRNA_i_^Met^. Non-aminoacylated tRNA_i_^Met^ has a K_d_ value of roughly 130 nM for both eIF2-GTP and eIF2-GDP, whereas “charged” Met-tRNA_i_^Met^ has a K_d_ of 9 nM for eIF2-GTP^61^. Reduced ternary complex as a result of too little Met-tRNA_i_^Met^ would be expected to reduce the number of ribosomes bound to mRNA, resulting in the observed decrease in the P/M ratio and number of polysome peaks. The hypothesis that mRNA translation initiation is suppressed due to a lack of ternary complex formation is supported by our previous findings that methionine deprivation in murine embryonic fibroblasts resulted in a GCN2-/eIF2α kinase-independent but ATF4-dependent increase in the integrated stress response^62^. The ability of methionine deprivation to increase the ATF4 level and the integrated stress response in the absence of eIF2 phosphorylation is most easily explained by methionine deficiency suppressing ternary complex formation by an alternative mechanism that does not involve eIF2 phosphorylation. This explanation is also supported by the findings of Dever *et al*.^63^ in *gcn2* knockout yeast. A reduction in the copy number of the initiator tRNA genes in the *gcn2* knockout yeast resulted in an increase in GCN4, the ATF4 analogue, in a manner dependent on an upstream open reading frame in the GCN4 mRNA. A lack of ternary complex is known to result in a decrease in global mRNA translation but an increase in ATF4 translation in mammals, and the increase in ATF4 abundance in turn is known to lead to an increase in the expression of genes that are activated by the binding of C/EBP-ATF4 heterodimers to C/EBP-ATF4 response elements on stress responsive genes^64^. In addition to reduced 43S PIC formation when Met-tRNA_i_^Met^ is deficient, a decrease in charged tRNA_i_^Met^ could possibly impair start site recognition by allowing uncharged tRNA_i_^Met^ to bind to eIF2, resulting in the formation of an ineffective 43S PIC. The expected result of either too little ternary complex or an ineffective ternary complex is that the 48S complex would be unable to form or to recognize the correct start site. Another possibility is that a decrease in ternary complex formation occurs due to a decrease in eIF2B guanine nucleotide exchange that is not dependent on eIF2 phosphorylation, as has been reported in liver of *Gcn2*-null mice fed a methionine-restricted, cysteine-free diet^36^.

Alternatively, methionine deprivation may interfere with methylation reactions that play an essential role in overall translation initiation. Methionine deprivation decreases the methylation of many macromolecules^65,66^, and methylation of DNA and histones has been shown to repress transcription^67^. Recent studies suggest that decreases in methylation due to methionine deprivation can suppress global mRNA translation and activate the integrated stress response independent of GCN2^60^. Working with MCF7 cells, Tang *et al*.^68^ demonstrated that methionine deprivation resulted in a transcriptional response that was different from that produced by a lack of other essential amino acids and that was dependent on the decrease in cellular SAM content. Recent studies showed that translation can be initiated independent of the 5′ cap through methylation of adenosine (m^6^A) in the 5′UTR, facilitating the binding of eIF3 to the m^6^A in the 5′UTR, leading to recruitment of the 43S PIC and initiation of translation independent of eIF4E^69,70^. Because methionine deprivation is known to reduce the extent of methylation reactions, we hypothesize that some of the changes in translation initiation observed in methionine-deficient HEK293T cells might be attributable to a decrease in m^6^A-dependent translation initiation.

In summary, we demonstrate that deficiencies of different amino acids suppress polysome formation and cell growth by different mechanisms and that at least some of these mechanisms may be independent of 4EBP1 binding to eIF4E or the phosphorylation of eIF2. Our results suggest that leucine and methionine each have major impacts on mRNA translation, apparently acting by quite different mechanisms, with leucine deprivation primarily inhibiting ribosome loading and methionine deprivation primarily impairing start site recognition. Further exploration of the effects of single amino acid deficiencies on mRNA translation may help elucidate mechanisms by which mRNA translation is regulated.

## Methods

### Cell culture and amino acid deprivation

Wild-type human embryonic kidney (HEK) 293T cells immortalized with SV40 large T antigen were cultured in high glucose Dulbecco’s modified Eagle’s medium (DMEM) (pH 7.4) supplemented with 1X nonessential amino acid mix, 100 units/mL penicillin and 100 µg/mL streptomycin (pen/strep), and 10% fetal bovine serum (FBS). Cells were maintained at 37 °C in an atmosphere of 95% air and 5% CO_2_. Cells were plated at a concentration of 4.0 × 10^4^ cells/cm^2^ and allowed to grow overnight before growth medium was replaced with experimental control or amino acid-deficient medium. Experimental control medium was the same as normal growth medium with the following changes: medium was prepared using DMEM that lacked sulfur amino acids, histidine, leucine and arginine (custom prepared by Gibco/Invitrogen) and was supplemented with 10% dialyzed FBS and l-cysteine, l-methionine, l-histidine, l-leucine and l-arginine to levels found in normal DMEM. Histidine-deficient (His-), methionine-deficient (Met-), leucine-deficient (Leu-) and arginine-deficient (Arg-) media were prepared similarly except the respective amino acids were not added back to the deficient DMEM. Cells were harvested after 12 h of culture in experimental medium. Final cell concentration was evaluated by measuring total protein using the bicinchonic acid (BCA) assay. Each figure contains data from a separate set of experiments; cell growth and polysome profiles were similar among all experiments.

### Puromycin labeling of proteins

Cells were plated as described above in 35 mm dishes. After 12 h of culture in treatment medium, 10 µM puromycin was added to the medium, and cells were harvested 10 min after the addition of puromycin. Cells were washed twice with ice-cold PBS and were then lysed in 100 μL of cell lysis buffer (50 mM Tris, pH 7.5, 150 mM NaCl, 1 mM EDTA, 0.5% v/v Nonidet NP-40) supplemented with Complete protease inhibitor and PhosSTOP phosphatase inhibitor (Roche). Cell lysate was mixed with an equal volume of 2X SDS PAGE loading buffer (0.1 M Tris-HCl, pH 6.8, 4% w/v SDS, 20% v/v glycerol, 200 mM dithiothreitol, and 0.05% w/v bromophenol blue). Proteins were separated by SDS-PAGE (10% w/v polyacrylamide) and were then transferred to Immobilon-P membranes. Membranes were blocked for 1 h in Tris buffered saline (50 mM Tris, 150 mM NaCl, pH 7.6) containing 5% w/v nonfat milk and 0.1% v/v Tween-20. Puromycin-labeled polypeptides were then quantified by incubating membranes with anti-puromycin (Developmental Studies Hybridoma Bank, #PMY-2A4) overnight at 4 °C and then with horseradish peroxidase-coupled secondary antibodies at room temperature for 1 h. Immunoblots were visualized using enhanced chemiluminescence and density was measured using ImageJ software. β-Actin antibody (Sigma-Aldrich) was used to quantify β-actin as a loading control.

### Polysome profiling

Cells were grown on 150 mm dishes as described above. At 5 min before cell collection, cycloheximide was added to the medium at a concentration of 100 µg/mL. Harvested cells were washed with ice-cold PBS containing cycloheximide (100 µg/mL). Cells were lysed in 600 μL of polysome lysis buffer (10 mM HEPES, pH 7.4, 100 mM KCl, 5 mM MgCl_2_) containing cycloheximide (100 µg/mL) and Triton-X100 (1% v/v). The lysate was centrifuged at 12,000 × g for 15 min at 4 °C. An aliquot was removed for western blotting, and 600 µl of supernatant was loaded onto 15–45% w/v sucrose density gradients, which were freshly prepared in SW41 ultracentrifuge tubes, followed by centrifugation at 32,000 rpm for 150 min at 4 °C in a SW41 rotor. Separated samples were collected from the top of the sucrose density at 0.375 mL/min through an automated fractionation system (Isco), and A_254_ values were continuously monitored and recorded. A_254_ profiles were scanned and traced (Inkscape). Polysome/monosome (P/M) ratios of the traced profiles were determined by aligning profiles to the bottom of the monosome peak and measuring the areas below the monosome and polysome peaks to a line equal to the lowest point on each analysis set.

### Western blotting

Protein concentrations of supernatant aliquots were determined by the BCA assay, and 50 µg protein per lane was added to NuPAGE LDS Sample Buffer supplemented with SDS and DTT (Invitrogen). Proteins were resolved using a NuPAGE 12% Bis-Tris Gel and NuPAGE MOPS SDS Running Buffer as described by the manufacturer. Proteins were transferred to Immobilon-P membranes, and membranes were incubated at room temperature with blocking buffer for near infrared fluorescent westerns (Rockland or Odyssey blocking buffer). The membranes were then incubated with antibody [caspase 3, cleaved caspase 3, 4EBP1, phospho-4EBP1 (Thr37/46), eIF4E, eIF2α, phospho-eIF2α (Ser51), or β-actin; 1:1000 dilutions; all from Cell Signaling] at 4 °C overnight. Membranes were then washed 3X in TTBS (50 mM Tris-Cl, pH 7.6, 150 mM NaCl, 0.1% v/v Tween 20) and exposed to secondary antibodies conjugated to AlexaFluor 680 or IRDye 800 (1:20,000, Li-COR Biosciences). Membranes were again washed 3X with TTBS, rinsed in PBS (pH 7.4), and the proteins were visualized and quantified using an Odyssey infrared imagining system (Li-COR Biosciences) and Odyssey software.

### eIF4E pull-down

Cells were cultured as described above in 150 mm dishes. Treated cells were washed 2X with PBS and then lysed in 1 mL of pulldown buffer [50 mM HEPES, pH 7.4, 50 mM NaCl, 2 mM EDTA, 2 mM EGTA, 50 mM β-glycerophosphate, 1 mM DTT, 1X Complete protease inhibitor (Roche) and 1X PhosSTOP phosphatase inhibitor (Roche) supplemented with 0.4% w/v NP-40]. Cell lysates were centrifuged at 10,000 × g for 15 min at 4 °C, and the supernatant was removed. For eIF4E-pulldown, 50 µL of γ-Aminophenyl-m^7^GTP (C_10_-spacer)-Agarose (Jenna Biosystems) was washed 2X with 500 µL of pulldown buffer without NP-40. After each wash the m^7^GTP-agaraose was centrifuged at 2,500x g for 1 min to pellet the beads, and the supernatant was discarded. Lysate containing 1 mg of protein was diluted to 500 µL in pulldown buffer and added to 50 μl of washed m^7^GTP-agarose. Samples were rotated at 4 °C for 90 min. After incubation, supernatant was removed and beads were washed 2X in 500 µL of pulldown buffer, followed by 500 µL of 0.5 mM GTP in pulldown buffer. Protein was eluted from the beads with the addition of 50 µL of 0.5 mM m^7^GTP in pulldown buffer and rotation at room temperature for 30 min. Incubation mixtures were then centrifuged at 2,500 × g for 1 min to pellet the beads, and the eluted protein in the supernatant were resolved by SDS/PAGE using a 10% (w/v) polyacrylamide gel. Protein transfer and visualization of eIF4E and 4EBP1 were done as described above.

### Plasmid generation and transfection

Plasmid pCMV6-Kan/Neo containing mouse *4ebp1* cDNA was purchased from Origene. Mutant 4EBP1(T37A/T46A) was generated using the Q5® Site-Directed Mutagenesis Kit (New England Biosystems). For 4EBP1 transfection, cells were transfected using the Lonza Nucleofector-2b device as described by the manufacturer. 1.0 × 10^6^ cells grown in control medium were combined with 5 µg of 4EBP1(T37A/T46A) plasmid, 4EBP1 wild-type (WT) plasmid or empty vector in Ingenio Electroporation solution (Mirus Bio). Cells were plated in normal growth medium in 100 mm culture dishes and were harvested 24 h post-transfection as described above. Protein (50 μg) was resolved by SDS-PAGE using a 12% (w/v) polyacrylamide gel. Protein transfer and visualization of 4EBP1 and phospho-4EBP1 (Thr47/46) were done as described above.

### Statistical analysis

Cell culture experiments were repeated three times, with cells grown up separately for each experiment. Results were expressed as fold of the mean for the same cell type cultured in control medium. Western band intensities, total protein concentrations and P/M ratios were analyzed using ANOVA and Tukey’s post hoc test (JMP 13). Differences were accepted at p ≤ 0.05. In experiments where there was no variance for the control (sufficient) condition due to normalization of results by the control value for each separate replicate of the experiment, the control group was not included in the ANOVA as this would violate the assumption of equal variance. Instead, 95% confidence intervals were used to determine whether values were significantly different than the control value.

### Ribosome profiling

Sucrose solutions were prepared in polysome buffer (10 mM HEPES, pH 7.4, 100 mM KCl, 5 mM MgCl_2_, 100 µg/mL cycloheximide and 2% v/v Triton X-100). Sucrose density gradients (15–45% w/v) were freshly prepared in SW41 ultracentrifuge tubes (Beckman) using a Gradient Master (BioComp Instruments). Cells were pre-treated with 100 µg/mL cycloheximide for 3 min at 37 °C followed by washing using ice-cold PBS containing 100 µg/mL cycloheximide. Cells were then lysed in polysome lysis buffer. For ribosome profiling without cycloheximide pretreatment, the same lysis buffer was used but cycloheximide was omitted. Cell debris were removed by centrifugation at 14,000 × g for 10 min at 4 °C. 500 µl of supernatant was loaded onto sucrose gradients followed by centrifugation for 2 h 28 min at 38,000 rpm at 4 °C in a SW41 rotor. Separated samples were fractionated at 0.75 mL/min through an automated fractionation system (Isco) that continually monitors A_254_ values.

Ribosome profiling was done as described previously^42^. Briefly, equal aliquots of the ribosome fractions separated by sucrose gradient were pooled and treated with *E. coli* RNase I (Ambion) to digest regions of mRNAs not protected by ribosomes. cDNA libraries were constructed from the ribosome-protected mRNA fragments by poly(A) tailing and reverse transcription using barcode-containing oligonucleotides (one for each treatment condition). For deep sequencing, the cDNA library was amplified by PCR using the Phusion High-Fidelity polymerase and primers containing Illumina cluster generation sequences. Equal amounts of barcoded samples for the three conditions were mixed together, and approximately 3–5 pmol of the pooled DNA sample was used for cluster generation followed by deep sequencing. Sequencing data are available at GEO (GSE112643).

To map the ribosome-profiling sequencing reads, adaptors were trimmed by Perl script, and the reads with length >35 nt or <25 nt were discarded. Low quality bases at the 5′ and 3′ ends of the reads were trimmed by Trimmomatic^71^ using parameters: AVGQUAL:28 LEADING:28 TRAILING:28 MINLEN:20. The trimmed reads were aligned to human transcriptome (GRCh38) using the software Bowtie^72^. No mismatch was permitted. The uniquely mapped reads were used to calculate ribosome densities along mRNA sites. In brief, for each ribosome P site (+12 nt offset from 5′ end of footprint) in an mRNA, a normalized ribosome density value was calculated by dividing the P-site read count by the average P-site read count across the coding sequence. All transcripts were aggregated by averaging each P-site position (relative to start codon or stop codon) across all the available transcripts embracing the P-site position. To avoid misinterpretations due to insufficient information, mRNAs with <32 mapped reads were excluded. To calculate frame ratio, ribosome footprints mapped to the coding region were stratified based on reading frames. Reads mapped to the first 100 nucleotides of the coding region were excluded from the frame ratio calculation, because highly elevated ribosome density in this region could cause bias to estimations of frame ratio. The reads corresponding to different frames (frame 1, 2 and 3) were calculated by dividing reads in frame 0, 1 and 2 by the total reads.

### Data availability statement

Sequencing data are available at GEO (GSE112643).
